# DNA methylation-based age estimation in pediatric healthy tissues and brain tumors

**DOI:** 10.18632/aging.202145

**Published:** 2020-11-09

**Authors:** Teresia Kling, Anna Wenger, Helena Carén

**Affiliations:** 1 Sahlgrenska Center for Cancer Research, Department of Laboratory Medicine, Institute of Biomedicine, Sahlgrenska Academy, University of Gothenburg, Gothenburg, Sweden

**Keywords:** DNA methylation, children, epigenetic clock, methylation age, brain tumor

## Abstract

Several DNA methylation clocks have been developed to reflect chronological age of human tissues, but most clocks have been trained on adult samples. The rapid methylome changes in children and the role of epigenetics in pediatric tumors calls for tools accurately estimating methylation age in children. We aimed to evaluate seven methylation clocks in multiple tissues from healthy children to inform future studies on the optimal clock for pediatric cohorts, and analyzed the methylation age in brain tumors. We found that clocks trained on pediatric samples were the best in all tested tissues, highlighting the need for dedicated clocks. For blood samples, the Skin and blood clock had the best correlation with chronological age, while PedBE was the most accurate for saliva and buccal samples, and Horvath for brain tissue. Horvath methylation age was accelerated in pediatric brain tumors and the acceleration was subtype-specific for atypical teratoid rhabdoid tumor (ATRT), ependymoma, medulloblastoma and glioma. The subtypes with the highest acceleration corresponded to the worst prognostic categories in ATRT, ependymoma and glioma, whereas the relationship was reversed in medulloblastoma. This suggests that methylation age has potential as a prognostic biomarker in pediatric brain tumors and should be further explored.

## INTRODUCTION

DNA methylation is part of the epigenetic regulation that determines which genes that should be expressed, and where, and this complex regulation is involved in embryogenesis, development and aging [1–3]. Aberrations in this system have been linked to multiple diseases, including cancer [4]. DNA methylation at certain loci changes with age [2, 5–8], and this was utilized a few years ago to create mathematical models to calculate the methylation age of a tissue of interest [9–11]. These so called methylation clocks are highly accurate in predicting the chronological age of humans [9–12], with a potential application in forensic analyses [13, 14]. Furthermore, it has been suggested that methylation age also captures the aspect of biological aging [15–17], i.e. why individuals of the same chronological age can appear older or younger, and differ in timing of functional decline, age-related diseases and death. Accelerated aging, higher methylation age (biological age) than chronological age, has for instance been demonstrated in patients suffering from the premature aging disorder Werner syndrome [18], Hutchinson Gilford progeria [19], in blood and brain tissue of individuals with Down syndrome [20], in cancer tissue [10, 11] and also to be predictive of cancer risk and all-cause mortality in healthy individuals [15, 21, 22].

The methylation pattern undergoes changes over the span of a life-time mainly by losing methylation with increasing age [23]. Changes in DNA methylation occur more rapidly in children than adults [24], with the majority of changes in childhood taking place during the first five years of life [25]. However, among the seven currently published and publicly available (as source code or R package) methylation clocks; Hannum [9], Horvath [10, 11], Epigenetic Timer of Cancer (EpiToc) [26], PhenoAge [27], Skin and blood clock [19], Pediatric-Buccal-Epigenetic (PedBE) clock [28] and Wu [29], only two of them (PedBE and Wu) have been focused exclusively on pediatric samples (buccal cells and blood respectively; Table 1). We therefore aimed to investigate and compare these seven methylation clocks to determine the most accurate one for pediatric cohorts of various tissue types, and to apply them for studies of brain tumors in specific. We were particularly interested in brain tumors since they are the leading cause of cancer-related deaths in children [30], and predictive/prognostic biomarkers are needed. One previous study showed age acceleration using the Horvath clock in the high-grade brain tumor glioblastoma (GBM) in a mixed cohort of pediatric and adult patients, and that the acceleration varied across subtypes [10, 11]. We hypothesized that methylation age would be accelerated, not just in high-grade tumors such as GBM, but in all pediatric brain tumors since DNA methylation is crucial during development and tumorigenesis, and significant for classification/subtyping of pediatric brain tumors [31–33]. Further, we theorized that acceleration would differ between the various diagnoses/subtypes and aggressiveness of the tumors and could thus be used as a prognostic biomarker. To evaluate this hypothesis, we investigated, in total, seven publicly available methylation clocks, 448 pediatric control samples from four tissue types, and brain tumors from 1434 children.

**Table 1:** Features of the publicly available methylation clocks.

**Methylation clock**	**Tissue types trained on**	**Age group of training samples**	**Type of methylation clock**
Hannum [9]	Blood	Adults (19-101 years)	Modelled to reflect chronological age
Horvath [10, 11]	Pan-tissue	Children and adults	Modelled to reflect chronological age
epiTOC [26]	Blood, 11 different fetal tissues (cord blood, liver, brain, heart etc.)	Adult blood (19-101 years), fetal tissues	Modelled to reflect mitotic-like clock approximating number of stem cell divisions
PhenoAge [27]	Blood	Adults (21-100 years)	Includes clinical biomarkers (glucose level, white blood cell count etc.) in addition to chronological age to select CpG sites and estimate the phenotypic/biological age
Skin and blood [19]	Blood, buccal, fibroblast, skin, epithelium	Children and adults (birth-85 years)	Modelled to reflect chronological age
PedBE [28]	Buccal	Children (birth-20 years)	Modelled to reflect chronological age
Wu [29]	Blood	Children (1-18 years)	Modelled to reflect chronological age

## RESULTS

### Few methylation clocks are suitable for pediatric samples of multi-tissue origin

**Figure 1:**
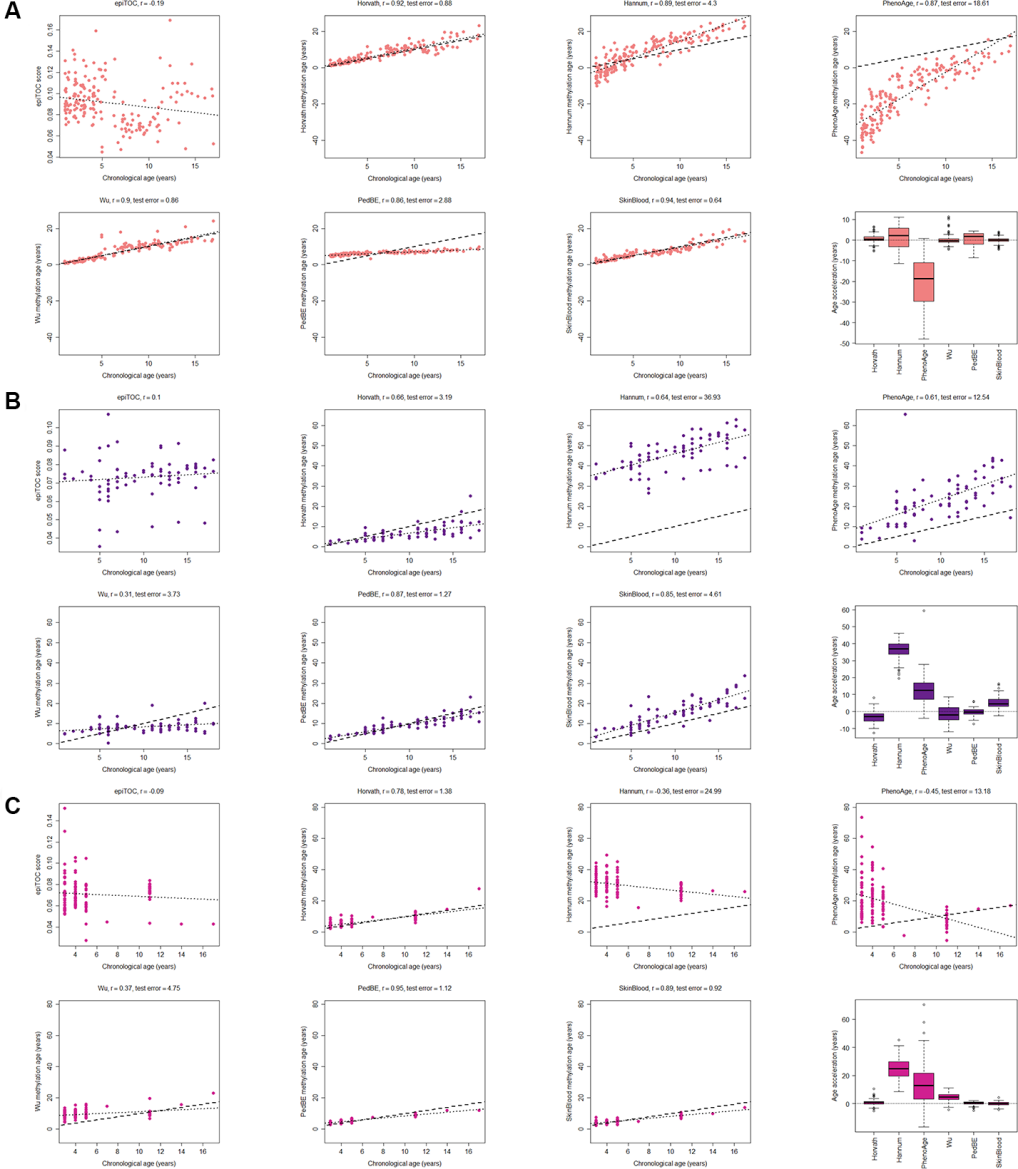
**Evaluation of seven methylation clocks in three tissues from healthy children.** (**A**) Comparison of seven methylation clocks in blood samples (n = 188) from healthy children with estimated methylation age (y-axis) vs chronological age (x-axis). The dashed line represents y=x (i.e. the estimated methylation age is the same as the chronological age), and the dotted line shows the chronological age to methylation age regression line. Pearson correlation between the methylation age and chronological age is indicated as r above each sub figure together with test error, which we define as the median difference in years between the methylation age and chronological age. The boxplots display age acceleration = methylation age – chronological age. The epiTOC score is calculated as an average Beta value over a set of 385 CpG sites which cannot be translated into an age estimate in years and we have therefore chosen to not include it in the boxplots or to display the dotted line or calculate the test error. (**B**) The performance of the seven clocks in buccal (n = 72) and (**C**) saliva (n=121) samples from healthy children.

The majority of the methylation clocks are trained exclusively or predominantly on samples of adult origin and we therefore first evaluated how the estimated methylation age correlated with the chronological age in children in three common and easily accessible tissues; blood, buccal cells and saliva (see age ranges in Supplementary Table 1). As expected, the clocks trained on data containing pediatric blood samples had the best correlation scores (r≥0.90) and the least deviation from the chronological age (age acceleration: Horvath mean = 0.72 years, standard deviation (sd) = 1.87; Wu mean = 0.17 years, sd = 2.1; Skin and blood mean = 0.011 years, sd = 1.41) using three datasets with blood from, in total, 188 children (Figure 1A). The correlation to chronological age for the Skin and blood clock was significantly better (adj. p<0.05) than the correlation for all of the other clocks. In contrast, clocks trained exclusively on adult blood samples had larger spread and deviated more from the chronological age (Age acceleration: PhenoAge mean = -20.6 years, sd = 10.6; and Hannum mean = 1.34 years, sd = 5.23), whereas epiTOC (trained on adult blood and fetal tissues) correlated poorly with the chronological age (r=-0.19) and the other clocks (r ranging between -0.41 and 0.04; Supplementary Figure 1A). This highlights the difference in aging in children versus adults, and the importance of using methylation clocks trained on pediatric samples.

A factor that could affect certain clocks is the need for adjusting the methylation age with a tissue-specific constant (intercept and slope), as reported for the Hannum clock [9]. We did not adjust the methylation age estimates, but instead analyzed the residual error in the clocks (i.e. the deviance between the estimated methylation age and the regression line), which is unaffected by the addition of constants, but the results were largely unchanged (Supplementary Figure 2A) for most clocks. However, the performance of for example the PedBE clock could be improved with this adjustment.

When analyzing methylation age, it is common to correct for the cell composition in blood, since different proportions of cell types may influence the methylation estimates from whole blood samples. Therefore, we investigated the sensitivity of the clocks to varying proportions of six cell types in blood; (CD8+) T cells, helper (CD4+) T, natural killer, B cells, monocytes and granulocytes, by estimating the cell type composition [34] in the above data. The analysis showed (Supplementary Figure 3) that depending on the choice of clock, correcting for cell type proportions could be needed, for example in the case of epiTOC that correlated significantly (adj. p <0.05) with the proportion of (CD8+) T cells (r = 0.71), natural killer cells (r=-0.26), B cells (r = 0.41) and granulocytes (r = -0.47). On the other hand, Horvath had no significant correlation to the proportion of any cell type, which is in line with previous results [10, 11].

Two of the clocks trained on buccal tissue (PedBE and Skin and blood) were superior to the others in terms of correlation to chronological age (test for difference in correlation p-value < 0.05 against each of the other clocks). There was no significant difference in correlation to chronological age between these two clocks, but the bespoke clock for buccal cells in children (PedBE) displayed less variation in the age estimates (residual se = 1.89, test for difference in standard error to Skin and blood p = 7.5e-08) (Figure 1B, Supplementary Figures 1B, 2B). Also, the Skin and blood clock overestimated the methylation age compared to the chronological age (age acceleration mean = 5.44 years).

Three clocks (Horvath, PedBE and Skin and blood) were the best performing clocks in saliva (Figure 1C, and Supplementary Figures 1C, 2C, test for difference in correlation p-value < 0.05 against each of the other clocks). Neither PedBE nor the Skin and blood clock were trained on saliva, but on buccal swabs, which have similarities to saliva as both are from the oral cavity and contains epithelial cells [35], likely explaining these results. There was a tendency for PedBE and Skin and blood to underestimate the methylation age for the older children (>10 years old), but larger cohorts are needed to verify this observation.

### Horvath multi-tissue clock is the most suitable clock for pediatric brain tissue

**Figure 2:**
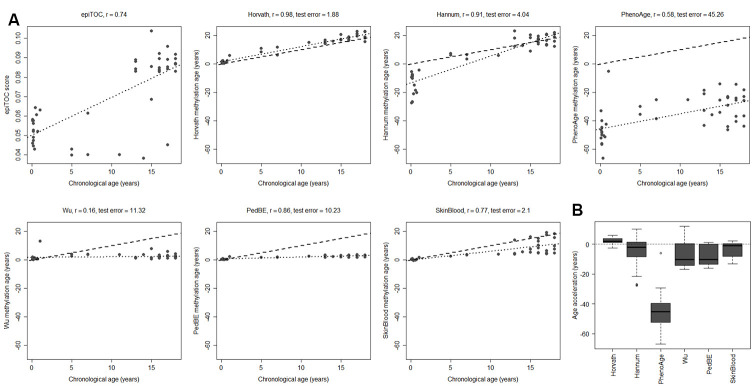
**Performance of seven methylation clocks in healthy pediatric brain tissue.** (**A**) Estimated methylation age (y-axis) vs chronological age (x-axis) in healthy brain tissue from 45 children for all investigated methylation clocks. The dashed line indicates y=x (a perfect correlation between methylation age and chronological age), and the dotted line displays the chronological age to methylation age regression. Test error is defined as the median absolute difference in years between the methylation age and chronological age. (**B**) Boxplots of age acceleration = methylation age – chronological age.

Having evaluated the most commonly used tissue types from children in DNA methylation studies, we next investigated brain tumors, since they are the leading cause of cancer-related deaths in children [30]. As a first step, we assessed the methylation clocks in a set of healthy pediatric brain tissue (n=45) to determine the most suitable clock for brain tissue in children (Figure 2 and Supplementary Table 1, Supplementary Figure 2D). The Horvath clock correlated the best with chronological age (r=0.98, test for difference in correlation adj. p < 1e-10 against each of the other clocks) and also had the least deviation from the chronological age (age acceleration mean = 2.19, sd = 1.86). Horvath slightly overestimated the methylation age compared to the chronological age whereas the majority of the clocks underestimated it. The PedBE clock had low variance in the age estimates (residual se = 0.57), but had, similar to the blood samples, an offset in the slope of the methylation age estimates vs the chronological age. Larger datasets would be needed to accurately estimate this slope to be able to improve the performance of the PedBE clock in brain and blood tissue.

**Figure 3:**
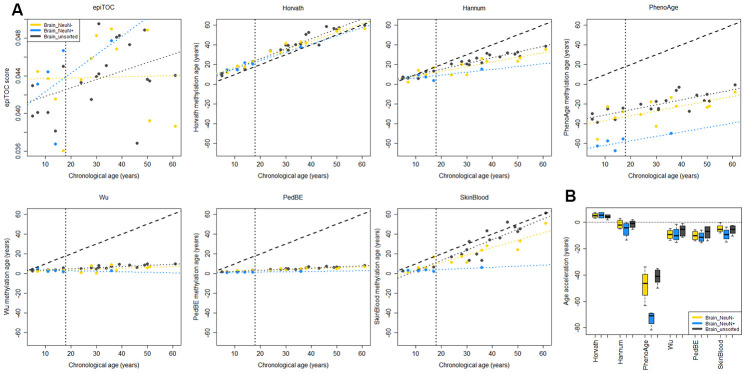
**Comparison of methylation clocks for sorted brain cells.** (**A**) The seven methylation clocks applied on matched samples from six children (≤18 years old) and 15 adults (separated by the vertical dotted line). Samples include unsorted brain tissue, and FACS sorted cells that are positive (NeuN+) and negative (NeuN-) respectively for a neuronal marker. The dashed diagonal line shows y=x, a perfect correlation between methylation age and chronological age, for reference, and the diagonal dotted lines display the regression lines of chronological age to methylation age for the three sample types. (**B**) Boxplot of age acceleration for the three sample types for the pediatric samples (boxplot for adult samples in Supplementary Figure 4). For the PhenoAge methylation clock, the NeuN+ cells have significantly lower age acceleration than NeuN- (adj. p = 0.024) and unsorted brain (adj. p = 0.0003).

To evaluate to what extent the methylation clock estimates are sensitive to different cell types in the brain, we used methylation data from matched tissues from six children and 15 adults in a previously published data set [36]. Apart from unsorted brain tissue, the data set included FACS sorted cells that were positive or negative for the neuronal marker NeuN (Neuronal Nuclei). Several of the clocks displayed a substantial intra-individual variation in methylation age between the cell types (Figure 3A). However, we could not detect any systematic differences, except for a significantly lower estimate for NeuN+ cells compared to NeuN- (adj. p = 0.024) and unsorted brain (adj. p = 0.0003) using PhenoAge for pediatric samples (Figure 3B), and for NeuN- cells compared to unsorted brain (adj. p = 0.038) using the Skin and blood clock on adults (Supplementary Figure 4). The Horvath clock was the most consistent in its estimation between cell types and since it also had the best correlation to chronological age and least deviation from it, as described above (Figure 2), we selected it for further studies on pediatric brain tumors.

### Horvath methylation age is accelerated in tumors and varies across diagnoses

**Figure 4:**
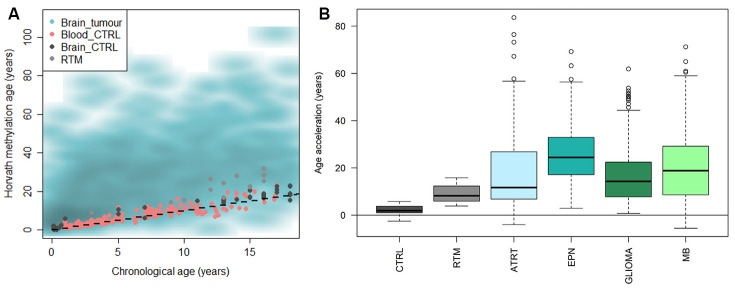
**Horvath methylation clock estimates of pediatric brain tumor samples in comparison to healthy blood and brain, and reactive tumor microenvironment.** (**A**) Horvath methylation age vs chronological age in pediatric brain tumors (n = 1112), healthy blood (n= 188) and brain tissue (n = 45) from children and reactive tumor microenvironment (RTM; n = 14) displaying an accelerated methylation age in a majority of the brain tumor samples. (**B**) The methylation age is significantly increased compared to normal brain tissue, in both RTM (adj. p = 1.1e-05) and four types of pediatric brain tumors; ATRT (adj. p = 1.3e-13), ependymoma (EPN; adj. p = <2e-16), glioma (adj. p = <2e-16) and medulloblastoma (MB; adj. p = <2e-16).

We applied Horvath’s methylation clock to a large data set of pediatric brain tumors (n=1112) [31] and also included a set of brain control tissue classified by DNA methylation as reactive tumor microenvironment (RTM; n=14), and investigated if the methylation age was increased compared to the chronological age (=accelerated aging). The results displayed that brain tumors were significantly accelerated compared to the chronological age (mean acceleration=19 years, range -11-85 years; adj. p-value < 2.2e-16), and also compared to blood (adj. p-value < 2.2e-16) and brain control tissue (adj. p-value < 2.2e-16) and RTM (adj. p-value = 1.3e-7) (Figure 4A). RTM samples also displayed significant age acceleration compared to chronological age (adj. p = 5.9e-07) and healthy brain tissue (adj. p = 6.7e-06).

The pediatric brain tumors were then classified and subtyped by DNA methylation according to the MNP classifier [31]. We first looked at four main diagnoses based on the classification; atypical teratoid rhabdoid tumor (ATRT), ependymoma, glioma and medulloblastoma. All tumor entities were significantly accelerated compared to the chronological age of the patients (adj. p < 1e-15), and also compared to the brain control tissue and RTM samples (Figure 4B and Supplementary Table 2). The age acceleration varied across the diagnoses and differed significantly (adj. p<0.01) between the groups in all cases except ATRT versus glioma, and ATRT versus medulloblastoma. We also noted that there was a large variance within each group.

### Pediatric brain tumors show subtype-specific accelerated aging

**Figure 5:**
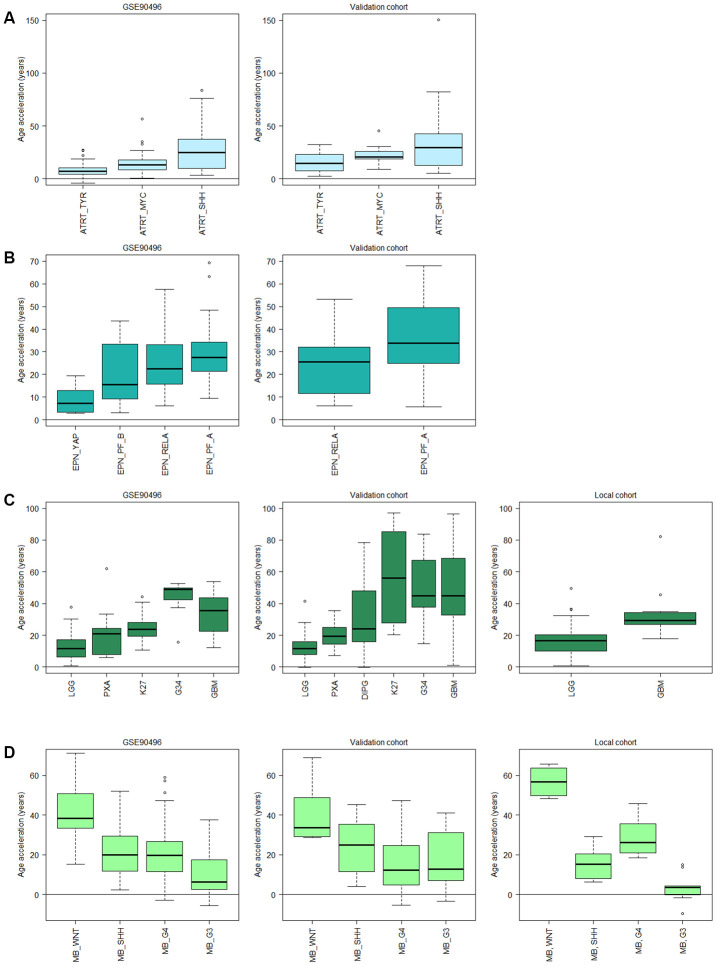
**Subtype specific age acceleration in pediatric brain tumors assessed by Horvath methylation clock.** (**A**) Age acceleration (Horvath methylation age minus chronological age) varies significantly (adj. p < 0.05) between all three ATRT subtypes in GSE90496 (n = 91; left panel), and the validation cohort (n = 49; right panel) shows the same trend with a significant difference (adj. p = 0.023) between the TYR and SHH subgroup. (**B**) In ependymoma (EPN), the YAP subgroup displays significantly lower age acceleration than the RELA (adj. p = 1.3e-6) and PF-A (adj. p = 1.0e-7) subgroups in GSE90496 (n = 157; left panel). Right panel with validation cohort (n = 65) shows significant difference between the RELA and PF_A subgroup (p = 0.02). (**C**) Left panel, GSE90496 (n = 295), is significantly different (adj. p < 0.05) for all pairs of gliomas except PXA (pleomorphic xanthoastrocytoma) vs LGG (low-grade glioma, mainly grade I) and K27 (diffuse midline glioma H3 K27M mutant), and GBM (glioblastoma) vs G34 (glioblastoma H3.3 G34 mutant). Middle panel with validation cohort (n = 153) varies significantly (adj. p < 0.05) between all pairs except K27 vs GBM and G34 and G34 vs GBM and DIPG, and right panel with local cohort (n = 86, p-value = 0.003). (**D**) The medulloblastoma (MB) subtypes in GSE90496 (n = 306; left panel) are significantly different for all pairs (adj. p < 1e-8) except SHH vs G4 (Group4). The age acceleration in the validation cohort (n = 48; middle panel) displays a similar trend in decreasing age acceleration, and in the local cohort (n=39; right panel) there is a significant difference between all pairs (adj. p < 0.05). Note that the subtypes in A-D have been ordered according to prognosis where the left-most subtype have the best prognosis and the right-most have the worst. Sample sizes for all included boxes is available in Supplementary Table 3.

Next, we used subtyping by DNA methylation within each diagnosis and examined whether age acceleration differed between subtypes. The ATRT subtypes; tyrosinase (TYR), myelocytomatosis oncogene (MYC), and sonic hedgehog (SHH), differed significantly (adj. p<0.05 in all comparisons), and the difference between SHH and TYR was validated (adj. p = 0.023) in another cohort of 49 patients (Figure 5A, Supplementary Table 3). The ATRT methylation subtypes were recently identified as independent risk factors [37], and the lowest risk group, TYR, corresponds to the least age-accelerated subgroup in our results. For ependymoma we also observed a trend of increased acceleration for the two subgroups (RELA and PF-A) that corresponds to the worst prognosis [38], and a significantly lower acceleration for the YAP subgroup with the best prognosis compared to the RELA (adj. p = 1.3e-6) and PF-A (adj. p = 1.0e-7) subgroups (Figure 5B). This indicates that methylation age could be used as a prognostic biomarker, and warrants further investigations in larger cohorts of ATRT and ependymoma.

We observed similar findings in glioma in multiple datasets (n=295, 153 and 86 respectively) where increased acceleration corresponded to increased tumor grade, and thus worse prognosis, starting with low-grade gliomas (LGG) of almost exclusively grade I, grade II pleomorphic xanthoastrocytoma (PXA) and then grade IV GBM subtypes having the highest acceleration (Figure 5C). The trend is the same in all three data sets, but the differences were not significant in all thus warranting further studies with larger cohorts. Interestingly, the age acceleration relationship was reversed in medulloblastoma as the subtype wingless integrated (WNT), which has the best prognosis, displayed the highest age acceleration while the most aggressive subtype, group 3 (G3) [39], had the lowest acceleration in two public datasets (n=15 and 33 respectively) and a local cohort (n=39; Figure 5D).

## DISCUSSION

The importance of epigenetics during child development, both in health and disease states, has made methylation age an interesting tool for studies of an epidemiologic nature as well as research on developmental disorders and processes in children. Studies so far on methylation age in children have shown acceleration associated with childhood trauma [40]**,** childhood abuse [41], childhood adversities [42], and threats and violence [43]. In addition, age acceleration has been associated with several childhood disorders such as autism [28, 29], asthma [44], and also faster pubertal development in girls [45], as well as increased body mass index [46]. However, the majority of the available methylation clocks today were trained purely or predominantly on adult samples, but Alisch et al. [24] have shown that methylation changes occur more rapidly in children than adults, suggesting that a methylation clock trained on adult samples might be inaccurate for pediatric samples. No study to date has performed a comprehensive comparison and evaluation of available clocks in the literature in pediatric cohorts. We therefore aimed to determine the most suitable methylation clock for children in various tissue types to inform future studies. Another objective was to evaluate methylation age as a prognostic biomarker in pediatric brain tumors. It has potential to be an additional tool for stratification of patients as DNA methylation has been used for tumor classification and subtyping (e.g. the brain tumor medulloblastoma) [31, 32, 39] and is now entering clinical diagnostic use [47, 48], and also as a biomarker for predicting treatment response (e.g. *MGMT* methylation in the high-grade tumor GBM) [49].

In this study, we included, to the best of our knowledge, all published clocks, available as R packages or as source code, for estimating methylation age, and compared them in different types of pediatric tissues that are easily accessible and commonly used. Their performance differed, presumably often as a consequence of what tissue type they were trained on. We observed that although the Horvath clock, trained on tissues from many sources, performed reasonably well in all our tested tissues, clocks trained exclusively on a specific tissue is slightly better in that context. For blood samples the Skin and blood clock slightly outperforms Horvath in our dataset, and the PedBE clock outperforms Horvath for buccal and saliva samples. This could be explained by the fact that PedBE is a clock designed solely for pediatric buccal samples, while Horvath was trained on samples of all ages and multiple tissues. Clocks that were trained, exclusively or partially, on pediatric samples proved the best in all tested tissues, and even outperformed the adult clocks in the cases where the clocks had been trained on the same tissue (i.e. blood). The results indicate that there is a need for methylation clocks trained solely on pediatric samples and preferably of a specific tissue type. Even though Horvath worked well for brain tissue, it overestimated the age of samples from healthy children, suggesting that a clock trained on only normal pediatric brain tissue could provide an improvement. The accuracy both in evaluation and training of clocks for small children could also benefit from documenting the chronological age in days or months instead of years.

Previous publications on methylation age in brain tumors have shown a median age acceleration of 35 years in a combined dataset of adult and pediatric GBM [10, 11], and accelerated aging was observed in 14 of 15 adult GBM (mean 25 years acceleration) and in all three evaluated meningioma tumors (mean 13 years acceleration) [50], which is in line with our findings in pediatric samples (median age acceleration 29 years in GBM local cohort). Also, a publication on adult glioma [51] showed that methylation age estimated with both Horvath and epiTOC differs between the subtypes of (adult) glioma, in line with our observations in pediatric samples of glioma and also in ATRT, ependymoma and medulloblastoma. As with gliomas, sub-types with the worst prognosis displayed the highest age acceleration in ATRT and ependymoma in our study. In medulloblastoma, however, we observed a reverse relationship between age acceleration and subtype, i.e. the WNT subtype with the best prognosis had the highest age acceleration. The correspondence between acceleration and prognosis suggests that methylation age might be of prognostic significance for ependymoma, ATRT, medulloblastoma, and glioma. However, larger methylation datasets with information on time to relapse and survival outcomes are needed to validate this finding.

A previous study [50] showed that Horvath methylation age in adult GBM and meningioma differed on average seven and three years respectively upon sampling from various locations of the tumors. How much the methylation age differs within pediatric tumors remains to be studied, but this potential intratumor variability could explain some of the variance in methylation age observed within the tumor subgroups. Cell-type heterogeneity within the brain could also contribute to a variance in the methylation age estimates, but we saw no significant difference in Horvath methylation age between unsorted healthy brain tissue, neuronal and non-neuronal cells, which is in line with what has previously been shown in a cohort predominantly consisting of adults [10]. We therefore did not adjust for cell-type heterogeneity in our analysis of healthy brain and brain tumors. Similarly, we show that methylation age estimated by the Horvath clock is not affected by varying proportions of blood cell types, as has previously been shown [10].

An issue with using public datasets, as we have done in this study, is that raw data is often lacking, and pre-processed and normalized data is instead more commonly available. The methylation data used in this study has therefore not been processed identically. McEwen et al. [52]**,** who investigated the Hannum and Horvath clocks with respect to normalization methods, reported that although the correlations with chronological age are unaffected, different normalization methods could lead to systematic offsets of the methylation age. This could be a factor contributing to the spread we see in our estimations. Additionally, McEwen et al. stated that the estimators are largely unaffected by the fact that not all CpG sites in the models are present on the newer Illumina EPIC arrays, suggesting that the clocks are robust to a small set of sites missing due to filtering. As for the joint analysis that we performed on samples from both Illumina 450K and EPIC arrays, the methylation values of CpG sites present on both arrays have previously been shown to highly correlate [53]. One potential source of error, however, might be due to the difference in quality in fresh frozen and FFPE samples. Although correlations between paired samples of the two preparation types are shown to be high [53], the risk of noise in FFPE samples is increased.

## CONCLUSIONS

This study compared seven published methylation clocks with available source code/R packages for blood, buccal, saliva and brain samples from healthy children, as well as pediatric brain tumor samples. The difference in performance between the clocks in these tissues are large, and not all of them are suitable for pediatric samples. Clocks that were trained solely or partly on pediatric samples performed the best. This can be explained by the faster rate of methylation changes in children than adults [24], and highlights the importance of using clocks trained on pediatric samples. The best clock for pediatric blood samples was the Skin and blood clock, while for saliva and buccal samples, PedBE was the most accurate with respect to correlation and deviation to chronological age. These findings will inform future studies on child development in health and disease states, as well as epidemiologic studies, in choosing the most accurate clock given their tissue type. The best clock for our area of interest, pediatric brain tumors, was Horvath’s multi-tissue methylation clock, even though it displayed a slight increase in methylation age compared to the chronological age also in healthy brain samples. With Horvath’s clock we showed that a majority of pediatric brain tumors have accelerated aging, and that it is subtype-specific in ATRT, ependymoma, medulloblastoma and glioma. Interestingly, we saw a relationship between the degree of acceleration in subgroups and prognosis, thus indicating that methylation age holds potential to be used for prognostication in pediatric brain tumors.

## MATERIALS AND METHODS

### Patients and samples

Tumor tissue was collected from pediatric brain tumor patients after signed informed consent by the parents. The study was approved by the Regional Ethical Review Board of Gothenburg (Dnr 604-12) and carried out in accordance with the relevant guidelines and regulations.

### DNA methylation analysis

DNA was extracted from fresh-frozen or FFPE tumor tissue and then bisulfite-modified (Zymo, Orange, USA), as previously described [53], and processed on EPIC methylation arrays (Illumina, San Diego, USA) at UCL Genomics (London, United Kingdom) according to the manufacturer’s protocols.

The raw DNA methylation data was processed using the statistical software R (https://r-project.org). Pre-processing and normalization (ssNoob) were done using R packages ChAMP [54, 55] (default filter settings, i.e. probes with less than 3 beads would be set to missing; probes with target CpG near a SNP and probes that align to multiple locations [56] are removed, as well as probes targeting the X and Y chromosomes) and minfi [57–59]. Only CpG sites present on both the 450K and EPIC array were kept for further analysis. Missing values were imputed using knn (K-Nearest Neighbor). Methylation profiles were classified by the online classifier MNP version 11b4 (https://www.molecularneuropathology.org/mnp) [31] using IDAT files.

Data from public datasets (GEO accession numbers according to Table 2) was downloaded as pre-processed and normalized Beta-values. Age at diagnosis (chronological age), annotated in years, was taken from Series Matrix files and/or Supplementary Tables of associated publications. Samples without annotated age were removed from analysis. As it is known that the methylation pattern differs between the different cell types of blood we estimated the cell type proportions of (CD8+) T cells, helper (CD4+) T, natural killer, B cells, monocytes and granulocytes in blood samples by Houseman’s reference-based method [34].

**Table 2:** Public datasets analyzed in this study.

**Sample type**	**GEO accession no.**	**Number of samples**
Blood_CTRL	GSE36054 [24]	134 children
	GSE111165 [36]	6 children
	GSE104812 [61]	48 children
Brain_NeuN-	GSE111165 [36]	4 children, 8 adults
Brain_NeuN+	GSE111165 [36]	4 children, 1 adult
Brain_unsorted/CTRL	GSE111165 [36]	6 children, 15 adults
	GSE44684 [62]	4 children
	GSE52556 [63]	14 children
	GSE41826 [64]	21 children
Buccal	GSE111165 [36]	5 children
	GSE109042 [65]	27 children
	GSE50759 [66]	40 children
Saliva	GSE111165 [36]	6 children
	GSE72556 [67]	95 children
	GSE110128 [68]	20 children
Reactive Tumor Microenvironment (RTM)	GSE90496 [31]	14 children
Brain tumours		
Mixed diagnoses	GSE90496 [31]	1112 children
Mixed diagnoses	GSE109379 [31]	129 children
ATRT	GSE70460 [69, 70]	17 children
ATRT	GSE141039 [37]	30 children
Ependymoma	GSE114523 [71]	52 children
Glioma	GSE50022 [72]	28 children
Glioma	GSE55712 [73]	35 children
Glioma	GSE77241 [74]	16 children
Medulloblastoma	GSE54880 [75]	15 children

### Methylation clocks and statistics

To calculate the methylation age, R packages cgageR (methylation clock: epiTOC [26]) and ENmix (methylation clocks: Hannum [9], Horvath [10, 11] and PhenoAge [27]) were used. The Wu methylation clock (age unit: months) was implemented as stated in Wu et al. [29]:

$$WuAge=F^{-1}(b_{0}+b_{0}CpG_{1}+\cdots +b_{111}CpG_{111}),$$

where

$$F^{-1}(x)=(toddler.age+1)e^{x}-1,\;\;\;\;\;\;\;\;\;\;\;\;if\;x\leq 0$$

$$F^{-1}(x)=(toddler.age+1)x+toddler.age,\;\;\;\;\;\;\;\;\;if\;x>0,$$

*toddler.age* = 48 months, and the regression coefficients *b_0_,…, b_111_* are defined in Supplementary Table 2 of [29].

We used online R code published at https://github.com/kobor-lab/Public-Scripts/ for PedBE [28], and R code available in the Supplementary methods of Horvath et al. [19] for the Skin and blood clock. Additional published methylation clocks, such as GrimAge [60], not openly available as source code or R packages were not evaluated here as they are not easily applied to large data sets.

Pearson correlation was used for all correlation calculations between chronological age and estimated methylation age. To test for difference between pairs of correlations, we used the Hotelling-Williams's Test. We define ‘age acceleration’ as ‘estimated methylation age’ minus ‘chronological age’ of the same sample. To test for significant difference (p-value < 0.05) between groups, we performed two-sample two-sided t-tests on age acceleration, assuming non-equal variance. Benjamini-Hochberg was used for multiple testing correction in all statistical tests.

## Supplementary Material

Supplementary Figures

Supplementary Tables
