# Supplementary material for: DNA methylation-based age estimation in pediatric healthy tissues and brain tumors
Source: Aging (Albany NY). 2020 Nov 9;12(21):21037–56. doi: 10.18632/aging.202145 (PMC7695434; doi:10.18632/aging.202145)
Supplement: Supplementary Figures [file aging-12-202145-s001..pdf]

SUPPLEMENTARY FIGURES

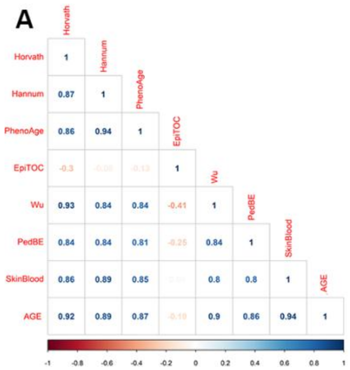

Supplementary Figure 1. Pearson correlations between methylation clocks and with chronological age in samples from healthy pediatric patients: (**A**) blood, (**B**) buccal, and (**C**) saliva.

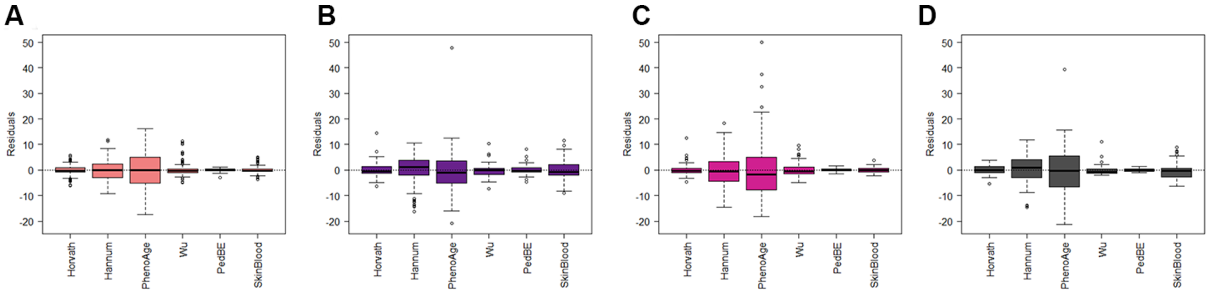

Supplementary Figure 2. Boxplots of residuals of the regression of methylation age to chronological age for samples from healthy children: (**A**) blood, (**B**) buccal, (**C**) saliva, and (**D**) brain.

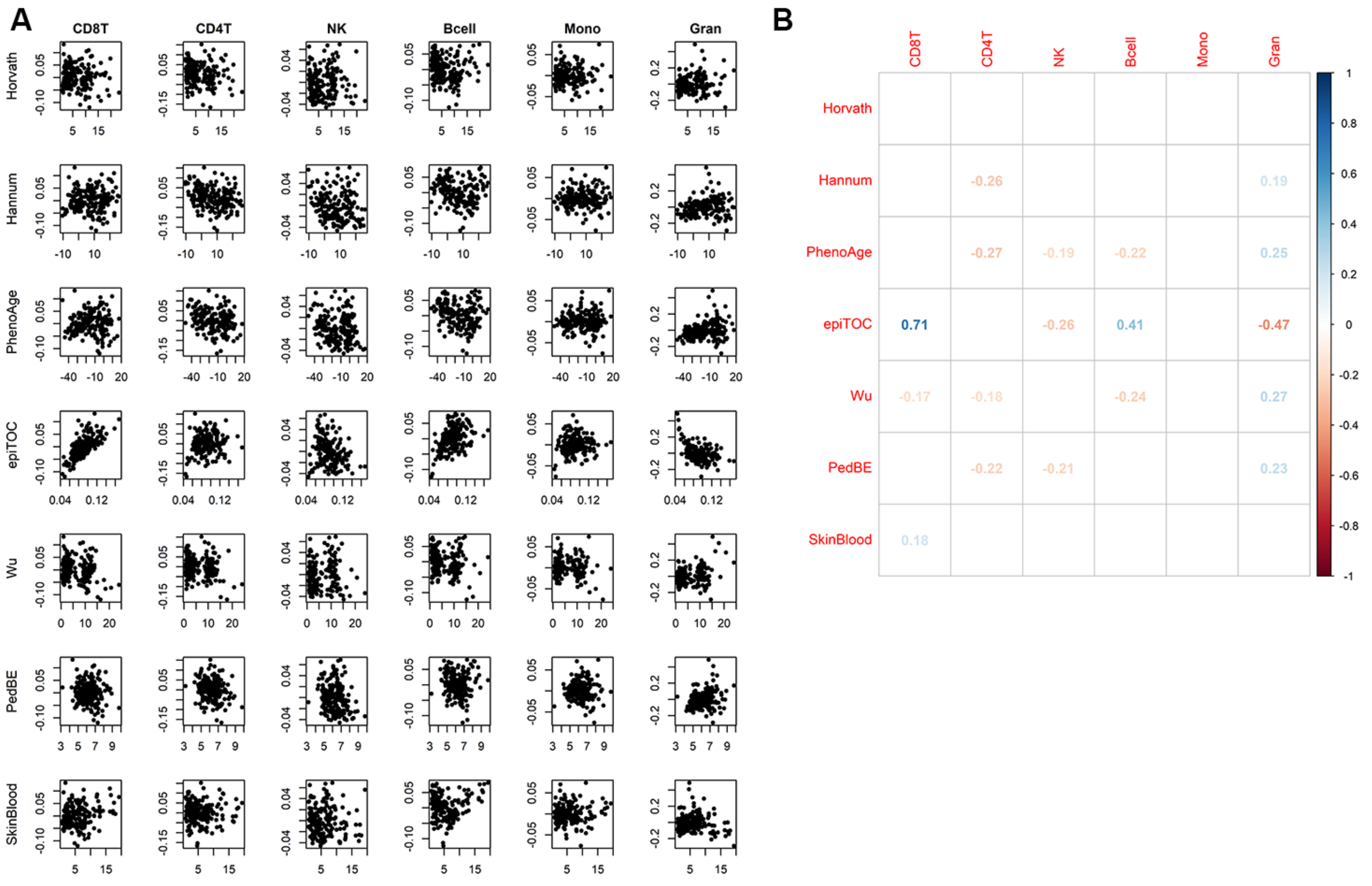

Supplementary Figure 3. (**A**) Scatter plots of age estimates from seven clocks (x-axis), against estimated proportions of six blood cell types, adjusted for chronological age: (CD8+) T cells (CD8T), helper (CD4+) T (CD4t), natural killer (NK), B cells (Bcell), monocytes (Mono) and granulocytes (Gran). (**B**) Pearson correlations of data from (**A**), where correlations with adjusted (Benjamini-Hochberg) p-values < 0.05 are displayed.

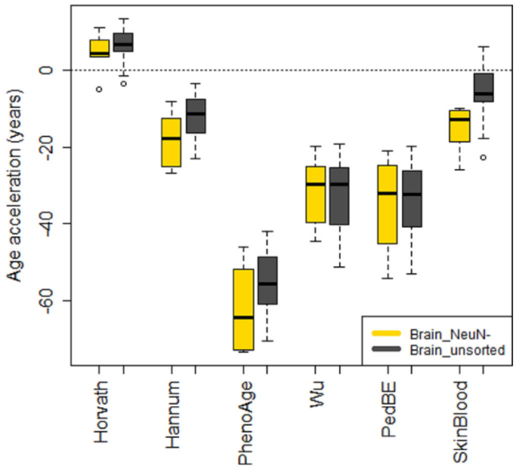

Supplementary Figure 4. Boxplots of age acceleration for two sample types for the adult samples. Samples include unsorted brain tissue, and FACS sorted cells that are negative (NeuN-) for a neuronal marker. For the Skin and blood methylation clock, the NeuN- cells have significantly (adj. p = 0.038) lower age acceleration than unsorted brain cells. FACS sorted cells positive for neuronal marker (NeuN+) are not included here as there were only one adult patient with methylation data for NeuN+ cells.
