# Supplementary material for: DNA methylation-based age estimation in pediatric healthy tissues and brain tumors
Source: Aging (Albany NY). 2020 Nov 9;12(21):21037–56. doi: 10.18632/aging.202145 (PMC7695434; doi:10.18632/aging.202145)
Supplement: Supplementary Tables [file aging-12-202145-s002..pdf]

## SUPPLEMENTARY TABLES

**Supplementary Table 1. Age ranges of samples from healthy individuals, for data plotted in Figures 1, 2.**

| | Min | Max | Median |
|---|---|---|---|
| **Blood** | 1 | 17 | 4.33 |
| **Saliva** | 3 | 17 | 4 |
| **Buccal** | 1 | 18 | 11 |
| **Brain** | 0.08 | 18 | 13 |

**Supplementary Table 2. P-values obtained from pairwise comparisons of age acceleration using t tests with non-pooled SD.**

| | CTRL | RTM | ATRT | EPN | GLIOMA |
|---|---|---|---|---|---|
| **RTM** | 1.1e-05 | - | - | - | - |
| **ATRT** | 1.3e-13 | 1.9e-05 | - | - | - |
| **EPN** | <2e-16 | 1.5e-14 | 0.0032 | - | - |
| **GLIOMA** | <2e-16 | 2.3e-06 | 0.2759 | 5.5e-12 | - |
| **MB** | <2e-16 | 9.8e-10 | 0.5542 | 8.5e-05 | 0.0017 |

P value adjustment method: BH.

**Supplementary Table 3. Number of paediatric brain tumour samples in boxplots in Figure 5.**

| Dataset | Sample type | Number of samples |
|---|---|---|
| **GSE90496** | ATRT TYR | 30 |
| | ATRT MYC | 22 |
| | ATRT SHH | 39 |
| **Validation cohort** | ATRT TYR | 18 |
| | ATRT MYC | 9 |
| | ATRT SHH | 22 |
| **GSE90496** | Ependymoma YAP | 10 |
| | Ependymoma PF B | 11 |
| | Ependymoma RELA | 52 |
| | Ependymoma PF A | 84 |
| **Validation cohort** | Ependymoma RELA | 18 |
| | Ependymoma PF A | 47 |
| **GSE90496** | LGG | 211 |
| | PXA | 13 |
| | K27 | 40 |
| | G34 | 8 |
| | GBM | 23 |
| **Validation cohort** | LGG | 55 |
| | PXA | 9 |
| | DIPG | 38 |
| | K27 | 16 |
| | G34 | 9 |
| | GBM | 26 |
| **Local cohort** | LGG | 73 |
| | GBM | 13 |
| **GSE90496** | Medulloblastoma WNT | 35 |
| | Medulloblastoma SHH | 72 |
| | Medulloblastoma G4 | 122 |

| | | |
|---|---|---|
| | Medulloblastoma G3 | 77 |
| **Validation cohort** | Medulloblastoma WNT | 6 |
| | Medulloblastoma SHH | 12 |
| | Medulloblastoma G4 | 20 |
| | Medulloblastoma G3 | 10 |
| **Local cohort** | Medulloblastoma WNT | 8 |
| | Medulloblastoma SHH | 6 |
| | Medulloblastoma G4 | 16 |
| | Medulloblastoma G3 | 9 |
